# Acetyl-coA synthetase 2 promotes acetate utilization and maintains cell growth under metabolic stress

**DOI:** 10.1186/2049-3002-2-S1-O9

**Published:** 2014-05-28

**Authors:** Zachary Schug, Barrie Peck, Dylan Jones, Qifeng Zhang, Israt Alam, Tim Witney, Elizabeth Smethurst, Shaun Grosskurth, Adrian Harris, Susan Critchlow, Eric Aboagye, Michael Wakelam, Almut Schulze, Eyal Gottlieb

**Affiliations:** 1 CR-UK The Beatson Institute, Glasgow, UK; 2 CR-UK London Research Institute, London, UK; 3 Weatherall Institute for Molecular Medicine, Oxford, UK; 4 The Babraham Institute, Cambridge, UK; 5 Imperial College, London, UK; 6 AstraZeneca, Macclesfield, UK; 7 Theodor Boveri Institute, Wurzburg, Germany

## 

During tumour formation and expansion, cancer cells encounter constantly changing environmental conditions in which nutrient and oxygen availability may be severely compromised. The metabolic transformation of cancer cells is characterised by distinct changes in metabolic activity that satisfy the exigencies of energy and biomass production imposed by continued cell proliferation. These metabolic adaptations often involve increased consumption and metabolism of extracellular nutrients, mainly glucose, amino acids and lipids. During periods of nutrient or oxygen deprivation, cancer cells can also modify their metabolism to adapt to these specific challenges. Here we report the results of a functional genomics study that revealed that the activity of acetyl-coA synthetase 2, an enzyme that converts acetate into acetyl-coA, contributes to cellular growth under oxygen and nutrient stressed conditions. ACSS2 was required to provide acetyl groups for lipid biosynthesis. Moreover, ACSS2 was essential for cancer cell growth and survival under physiologically relevant growth conditions and its depletion blocked tumour growth *in vivo*. In summary, our data demonstrate a previously unappreciated role for acetate as a nutritional source for the growth and survival of breast and prostate cancer cells under metabolic stress.

